# The Significance of the ProtDeform Score for Structure Prediction and Alignment

**DOI:** 10.1371/journal.pone.0020889

**Published:** 2011-06-28

**Authors:** Jairo Rocha, Ricardo Alberich

**Affiliations:** Department of Mathematics and Computer Science and IUNICS, University of the Balearic Islands, Palma, Spain; Semmelweis University, Hungary

## Abstract

**Background:**

When a researcher uses a program to align two proteins and gets a score, one of her main concerns is how often the program gives a similar score to pairs that are or are not in the same fold. This issue was analysed in detail recently for the program TM-align with its associated TM-score. It was shown that because the TM-score is length independent, it allows a P-value and a hit probability to be defined depending only on the score. Also, it was found that the TM-scores of gapless alignments closely follow an Extreme Value Distribution (EVD).

The program ProtDeform for structural protein alignment was developed recently and is characterised by the ability to propose different transformations of different protein regions. Our goal is to analyse its associated score to allow a researcher to have objective reasons to prefer one aligner over another, and carry out a better interpretation of the output.

**Results:**

The study on the ProtDeform score reveals that it is length independent in a wider score range than TM-scores and that PD-scores of gapless (random) alignments also approximately follow an EVD. On the CASP8 predictions, PD-scores and TM-scores, with respect to native structures, are highly correlated (0.95), and show that around a fifth of the predictions have a quality as low as 99.5% of the random scores. Using the Gold Standard benchmark, ProtDeform has lower probabilities of error than TM-align both at a similar speed. The analysis is extended to homology discrimination showing that, again, ProtDeform offers higher hit probabilities than TM-align. Finally, we suggest using three different P-values according to the three different contexts: Gapless alignments, optimised alignments for fold discrimination and that for superfamily discrimination.

In conclusion, PD-scores are at the very least as valuable for prediction scoring as TM-scores, and on the protein classification problem, even more reliable.

## Introduction

The great availability of protein classifiers is motivated by several circumstances: the growing number of protein structures in the PDB [1], the still unknown gold score for protein classification [2] and the lack of a structural aligner with low error probabilities. These needs prompted us to develop an aligner, ProtDeform [3], a model and algorithm for protein comparison using a sequence of local rigid transformations to find proper alignments to match the structures. It thus allows a non-rigid deformation of one protein over another, proposing flexible alignments.

We have successfully proved that ProtDeform is one of the best structural classifiers including Dali [4]; Matras [5]; MATT [6], PPM [7]; SSAP [8]; Rash [9] and TM-align [10] on benchmarks based on standard protein classifications (CATH [11] as well as SCOP [12]) and hand curated alignments (SISYPHUS [13]). One issue addressed in this paper is the change we have made in the ProtDeform score, *PD-score*, in order to increase the speed of the system by making the calculations coarser.

Recently, Xu and Zhang [14] demonstrated that the TM-score of gapless alignments is length independent and closely follows an Extreme Value Distribution (EVD), results useful for calculating the TM-score statistical significance of folding models with respect to native structures. Also proposed in the same study was a TM-score of approximately 0.5 as a threshold to decide whether two structures are of the same fold or not, since it is the score above which the probability of two proteins being in the same fold is above 0.5, and below which, the probability is below 0.5.

In this paper we extend the previous analysis to the ProtDeform score and to homology discrimination. We test whether the PD-scores for gapless alignments approximately follow an EVD and then examine the length independence of both scores as calculated by structural aligners. We determine score thresholds for both fold and homology discrimination and make a statistical significance calculation more appropriate for both fold and homology decisions when an optimisation program is used. We then compare the performance of ProtDeform with respect to TM-align. In short, we tell researchers how to better interpret the PD-scores and TM-scores.

When posed with the problem of defining the significance of scores on a recognition test, first of all, we need to define what kind of scores we expect to see on objects that should not be recognized as similar. Levitt et al [15] give a common framework for sequence and structure protein score significance; they suggest that the baseline level of structural similarity seems to be that seen between domains of different classes. We believe that we should specify the context of application of the scores. Let's assume that a researcher would like to know if the domains in a protein pair are in the same fold and she gets a score from a TM-align or ProtDeform. She would like to know the statistical significance of the score, meaning the probability of obtaining the score assuming that the domains do not appear in the same fold. Clearly in this context the baseline level must be that which is seen between domains of different folds as calculated by the program. In contrast, if the researcher gets the score from the known corresponding residues between a folding model and its native structure in order to judge if they are similar, i.e. have the same fold, she would like to know the probability of obtaining the score, assuming that structures are of the same size, trivially aligned and not from the same fold. Obviously, for the latter case, scores for gapless alignments between chains of the same size and not in the same fold should be considered as the baseline level scores. We shall analyse both cases; in other words, we will propose different significance values depending on whether the alignments stem from folding or from program aligners.

A public server for the program can be found at http://bioinfo.uib.es/~recerca/ProtDeform. The program is also available for free download. The data related with this study is at http://bioinfo.uib.es/~recerca/ProtDeform/PDscore.

## Results

First, we demonstrate that the scores are not truly length independent except in the range corresponding to medium-to-high hit probabilities. Then, we calculate several P-values for different test situations and, finally, we calculate a posteriori hit probabilities and discuss an application.

We consider two different situations dealing with a pair of domains. In the first one, a score is calculated between an experimental structure and a predicted model for the gapless identity alignment in order to ascertain if the model and the structure are found in the same fold or not. In the second one, a score for the best alignment is determined by a program in order to know if two domains are in the same fold or not. We then carry out a score analysis for each different population.

### The length independence of the scores

We know that several scores used for protein structural comparison are not length invariant, such as RMSD, the Dali Z-score, the MAMMOTH score and others [14], [16]. In contrast, the TM-score is length invariant, i.e. the magnitude of the TM-score for random protein pairs is protein size independent [14], [17]. As a consequence of this invariance, the P-value can be expressed as a sole function of TM-score.

**Figure 1:**
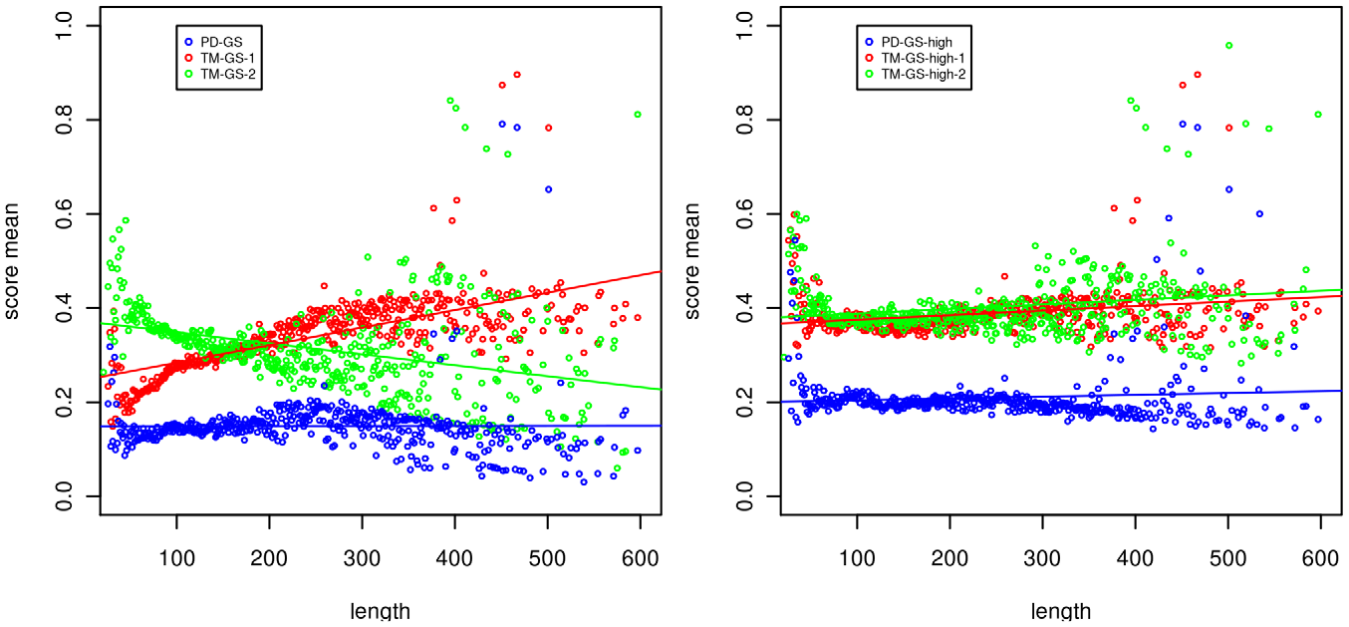
Average scores for the set GS in terms of the protein length. Left: The average score depends on the length of the first domain and on the second domain for TM-align while ProtDeform is independent. Right: If only scores above the global average are considered, the dependency is insignificant. See Table 1 for the values of coefficient and the quality of the fitting. The displayed lengths below 600 cover 99.88% of the pairs.

Xu and Zhang confirmed that the TM-align score is length independent, as revealed in the original paper of Zhang and Skolnick [17]. However, what they showed is that the TM-score is invariant with respect to the minimum length of the target and query structures. We set out to determine whether the TM-align score was independent with respect to the target length in order to know if in a database search there was a bias towards long structures. We found that the TM-score calculated by TM-align is significantly and positively dependent on the length of the first parameter (model or query structure) and significantly and negatively dependent on the length of the second parameter (native or target structure), as shown in Table 1 and Figure 1. This holds true for both benchmark sets. The result reminds us that the TM-score is highly asymmetrical on the order of the two domains. Also, for TM-scores calculated for alignments done by ProtDeform, the dependency is still observable demonstrating that the TM-score is by itself length dependent. For the gapless tests, the length independence is not crucial because all predicted models for a native structure have the same length. However, it is worth noting that the TM-score is length independent for gapless alignments while the PD-score is not, which has some implications for the P-value calculation that we discuss in the next section.

**Table 1:** Average score regressions as a function of domain length weighted by length frequencies.

Test	length coef. 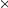 1000	adjusted 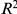
PD-XZ	−0.077	0.321
PD-XZ-high	0.002	−0.002
PD-GS	−0.004	−0.002
PD-GS-high	−0.056	0.181
TM-PD-GS-1	−0.128	0.460
TM-PD-GS-2	−0.185	0.479
PD-gapless	**0.370**	**0.932**
TM-XZ-1	**0.378**	**0.788**
TM-XZ-2	**−0.480**	**0.946**
TM-XZ-high-1	0.073	0.451
TM-XZ-high-2	−0.007	0.001
TM-symm-XZ	−0.054	0.285
TM-symm-XZ-high	−0.019	0.007
TM-GS-1	**0.491**	**0.790**
TM-GS-2	**−0.390**	**0.584**
TM-GS-high-1	0.119	0.304
TM-GS-high-2	0.028	0.007
PD-TM-GS-1	**0.263**	**0.576**
PD-TM-GS-2	−0.304	0.486
PD-TM-GS-high-1	−0.185	0.447
PD-TM-GS-high-2	0.004	−0.002
TM-gapless	0.039	0.180

The first column identifies the test: “high” means with scores above the average (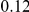 for PD and 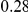 for TM); TM-PD means that PD-scores are calculated for the alignments done by TM-align; 1 or 2 means the first or second domain length, respectively; “symm” means taking a symmetrical score. The second column is the length coefficient multiplied by 1000, so it is the average score variation for 1000 amino acids. The third column is the adjusted 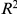. We can see in bold all the tests with a significant linear dependency (i.e., adj. 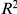 above 0.5).

The score-to-length ratio is such that in a domain database search, there is an expected 0.24 score decrease for a 500-amino-acid length increase in the XZ set, for instance. A change of 0.24 is meaningful only if it crosses the TM-score of 0.5 because as shown in [14] a phase transition takes place there in the a posteriori probability for fold similarity. This length dependency could affect not only the decision of whether two domains belong to the same fold or not but also the ranking of the most similar domains to a query. In other words, the TM-score decrease due to length dependency affects only domain pairs with TM-scores that are not too low. Because of this, the length dependency on pairs with low scores has no importance. When only the domain pair TM-scores above any threshold greater than the mean (0.28) are considered, the linear length dependency is no longer significant on any of the benchmarks.

In short, the TM-score is length dependent on both domains but particularly on non similar domains. In the most important medium-to-high score range, the linear dependency is negligible. Also, if a symmetrical TM-score is defined using the average of the evaluation of the two domain orders, the linear dependencies are eliminated.

For the PD-score case, the analysis proves simpler because of the symmetry of the score. The dependency of ProtDeform and its score on the length is not significant on any test. The same also can be said for domain pairs with medium-to-high scores (e.g., above 0.12). There is some length dependency in the PD-scores calculated for alignments computed by TM-align, as shown in Table 1. For gapless scores, there is a high positive dependency although, as we said before, it has no practical importance because the length of the predictions remains constant.

The conclusion of this section is that the scores are length linear independent in the discriminant range so the hit probabilities can be expressed depending only on the score. The ProtDeform score is length independent on the full score range. The PD-score for gapless alignments is length dependent but, as we shall discuss in the next section, it can be disregarded.

### The significance of the scores

**Figure 2:**
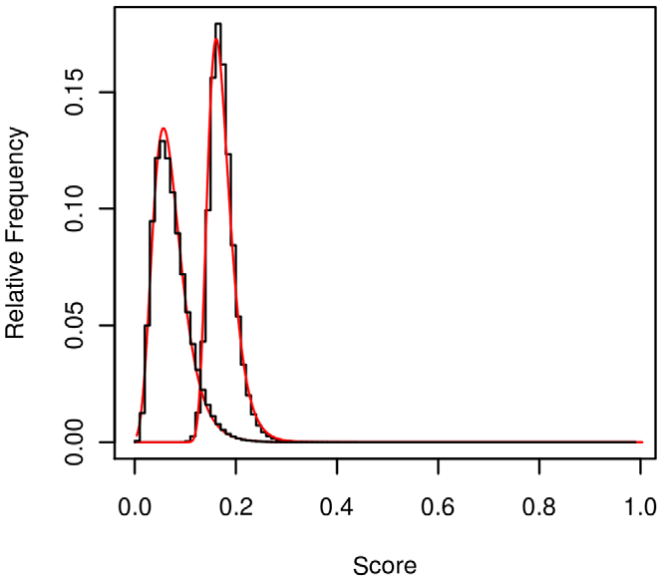
PD-score (mode on the left) and TM-score (mode on the right) distributions of gapless alignments for the XZ-diff-fold set. The lines represent the best approximations of Gumbel distributions found by R using fitdist: the parameters (location, scale) for PD-scores and TM-scores are, respectively, (0.058,0.027) and (0.162,0.021). For both scores, the coefficient of determination of the expected vs observed frequencies is 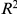 = 0.995. The score axis is for both PD-scores and TM-scores, although their values are not related.

For the case of gapless alignments, we assume that the population is the set of gapless alignments made between prediction and native complete domains of the same length. Since this population rarely occurs in nature, we consider the benchmark of gapless alignments between two domains, sliding the shorter one over the longer one. The null hypothesis for this case is that the two domains of equal length belong to different folds. The distribution of these scores is fitted to an EVD (Gumbel), as seen in Figure 2. Therefore, we confirmed on a different benchmark the close approximation of TM-scores to an EVD, and showed that PD-scores also closely follow an EVD. The location and scale reported by Xu and Zhang [14] was 0.151 and 0.024, respectively, for a different but analogous set.

**Figure 3:**
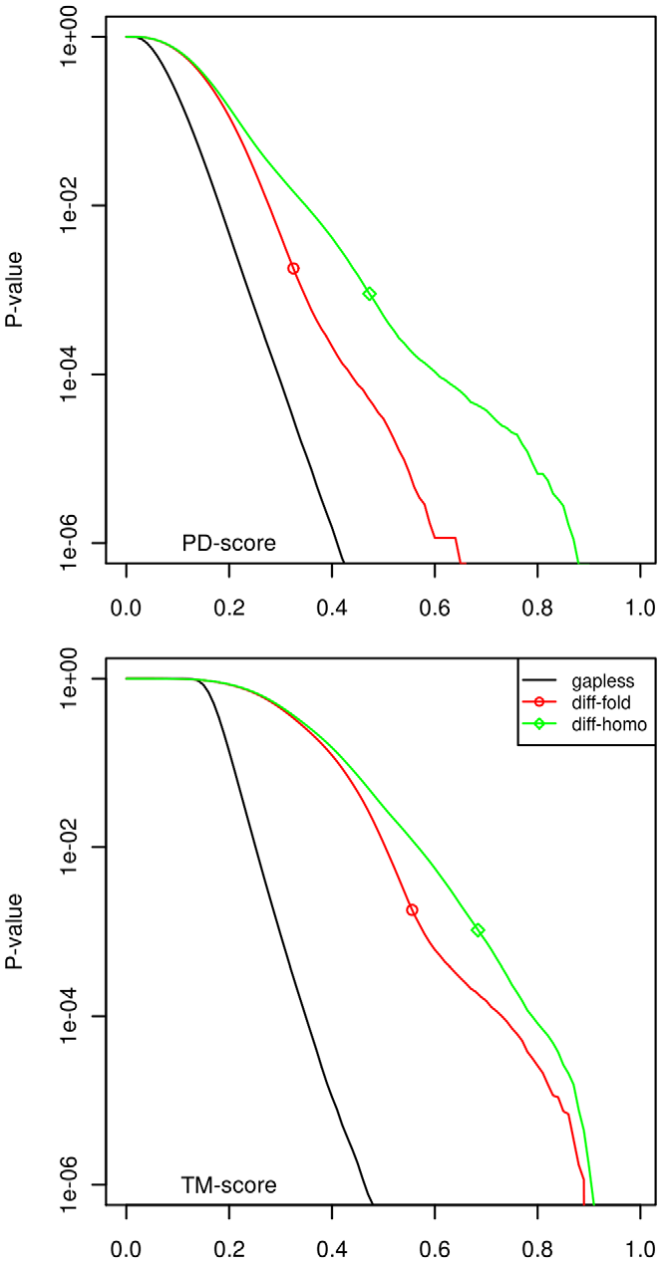
P-values when calculated for gapless and optimised alignments for the different fold and homology cases. Top panel: PD-scores; bottom panel: TM-scores. The round (diamond) point marks the score above which pairs can be considered in the same fold (homology). We can see that the P-values are different according to the three problems: prediction quality, fold discrimination and homology discrimination.

The PD-score mode is located 0.1 to the left of the TM-score one, with a similar scale, and, in general, the PD-score values are lower than the TM-score values. In Figure 3 we can see, for instance, that the probability of finding a TM-score greater than or equal to 0.27 among gapless alignments is lower than 0.005; for PD-scores, at the same level of significance, the score is 0.20.

**Figure 4:**
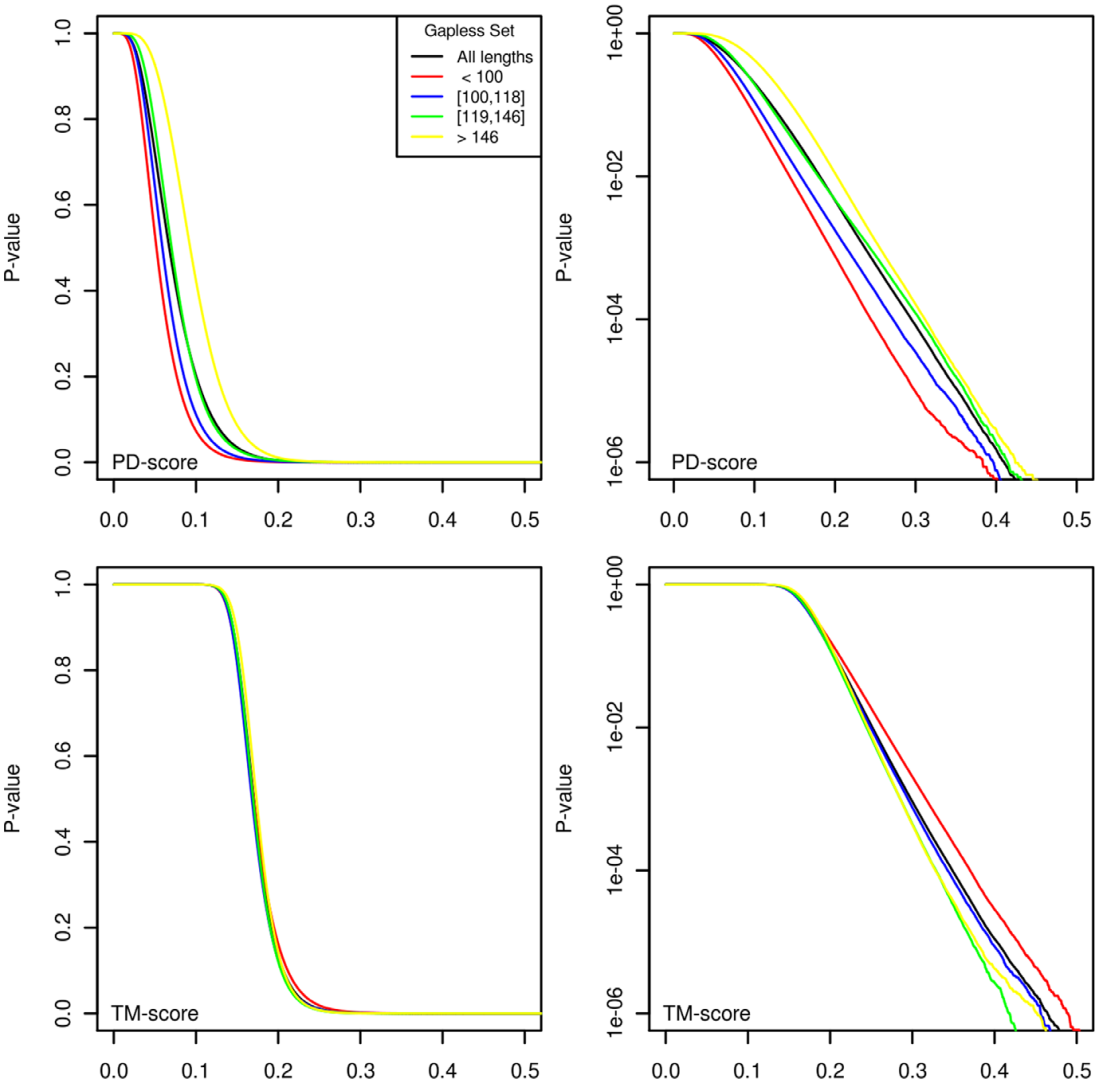
Gapless P-values according to the length. Top panels: PD-scores; bottom panels: TM-scores. We can see that length dependency is appreciable for PD-scores under 0.2, a non-discriminant range. Right panels: the logarithmic scale shows that the differences depending on lengths decrease exponentially with the scores above 0.2. The P-value curve for all lengths, therefore, is the only one used.

Since the PD-scores for gapless alignments are length dependent, according to Table 1, the P-values can be better expressed as a function of length. To see its real influence, we divided the scores according to the length into four sets of approximately equal size, with lengths in the intervals 1–99, 100–118,119–146,147-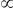. Figure 4 illustrates that the P-values increase a bit with the length within the unimportant low score range (below 0.2). The P-values for TM-scores are almost length independent. For the high scores, the logarithmic figures show that the P-values decrease exponentially with the length so it is not worth having different evaluations according to length.

**Figure 5:**
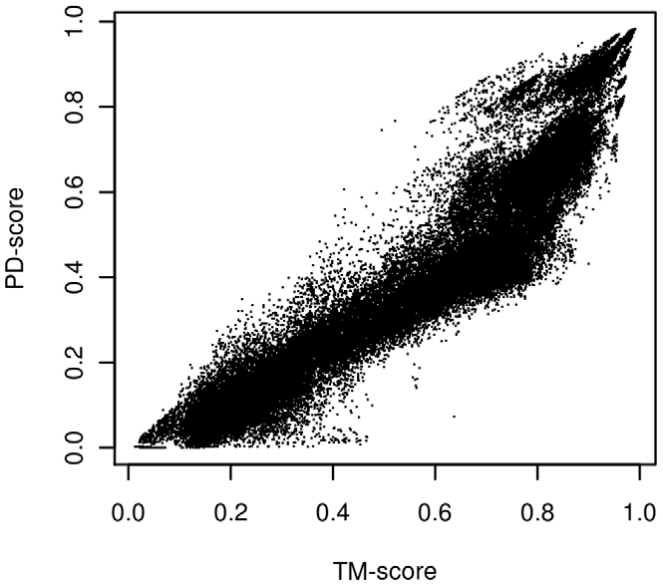
TM-scores vs PD-scores for the 70,964 models submitted to CASP8 with respect to their native structures. The Spearman correlation coefficient is 0.95, ample proof of agreement on the ranking most of time.

**Figure 6:**
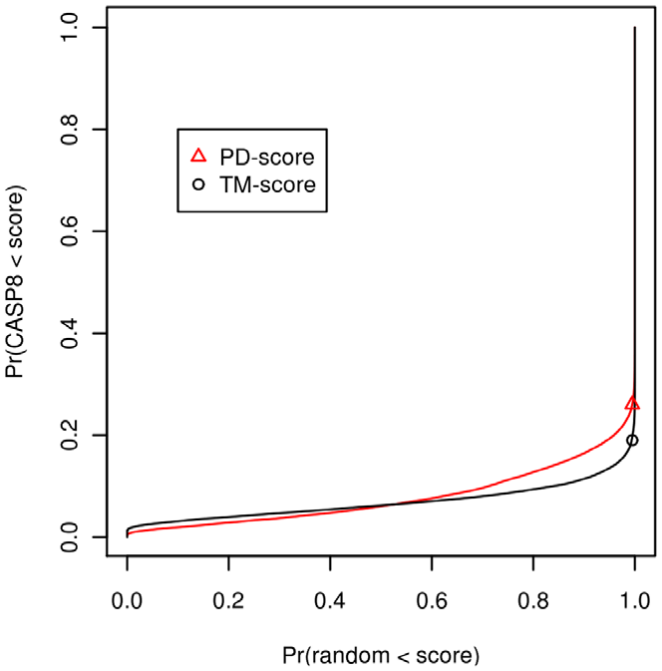
For each score 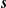, the curves show the fraction of the random (gapless) pair scores under 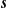 versus the fraction of the CASP8 prediction pair scores under the same 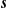. The marks correspond to the places for 99.5% of the random scores (P-value = 0.005). We can find the proportion of predictions below any given quality.

Real data for the case of scores between native structures and predicted models comes from CASP8. Over the 70,000 models calculated by the competitors, the Spearman correlation coefficient between TM-scores and PD-scores is 0.95, which shows that both scores would produce similar rankings, as seen in Figure 5. We can also apply the earlier significance analysis to see in Figure 6 that according to the PD-scores and TM-scores, 26% and 18%, respectively, have score values in the lower 99.5 percentile of the random scores. In other words, around a fifth of the predictions have a quality as low as 99.5% of the random scores. It should be added that PD-scores are somewhat more critical than TM-scores with the predictions.

We can see in Table 2 that for 14 out of 164 native structures (8.5%), 80% of the predictions have PD-scores as low as 95.5% of random pairs, and 8 structures also have 90% of their predictions as off as random ones. Also, we can see that for any native structure, there is at least one prediction better than a random one. The number of poor predictions according to the TM-score is lower, as we mentioned in the previous paragraph.

**Table 2:** Number of targets that have a given percentage of predictions with a score as low as 95.5% of the random scores.

poor	PD	PD	TM	TM
prediction %	number of targets	target %	number of targets	target %
50	28	17.07	18	10.98
60	22	13.41	13	7.93
70	18	10.98	9	5.49
80	14	8.54	5	3.05
90	8	4.88	1	0.61
100	0	0.00	0	0.00

For instance, 8 native structures (4.88%) have 90% of their predictions with PD-scores as low as random scores.

For the case of alignments found by an optimisation program for fold discrimination, we assume that the population is the set of alignments calculated by the program aligner. The null hypothesis for this case is that the alignment is between two domains not occurring in the same fold, represented by 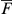. If the program gets a score 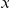, the P-value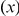 is the probability 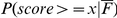. In our tests, we calculated P-values for ProtDeform and TM-align and show them in Figure 3, using the scores calculated for the set GS-diff-fold. To give an example, the significance of a TM-score of 0.556 is P-value(TM-score = 0.556) = 0.001799 and the significance of a PD-score of 0.325 is P-value(PD-score = 0.325) = 0.001791 according to the GS-sets. For homology discrimination, the P-values are also shown in Figure 3 for the GS benchmark.

As for the gapless case for PD-score, the TM-scores from alignments are length dependent, so the P-values would be better described with this additional parameter. However, the differences in the discriminant range are small, so we also use only one P-value curve for TM-align.

### The posterior probability of the scores

In this section we calculate the posterior probability that two domains are or are not in the same fold (or homology) given a score. This will help researchers judge whether the query domains are similar or not once they have a score. To do so, we use the two benchmarks already mentioned.

**Figure 7:**
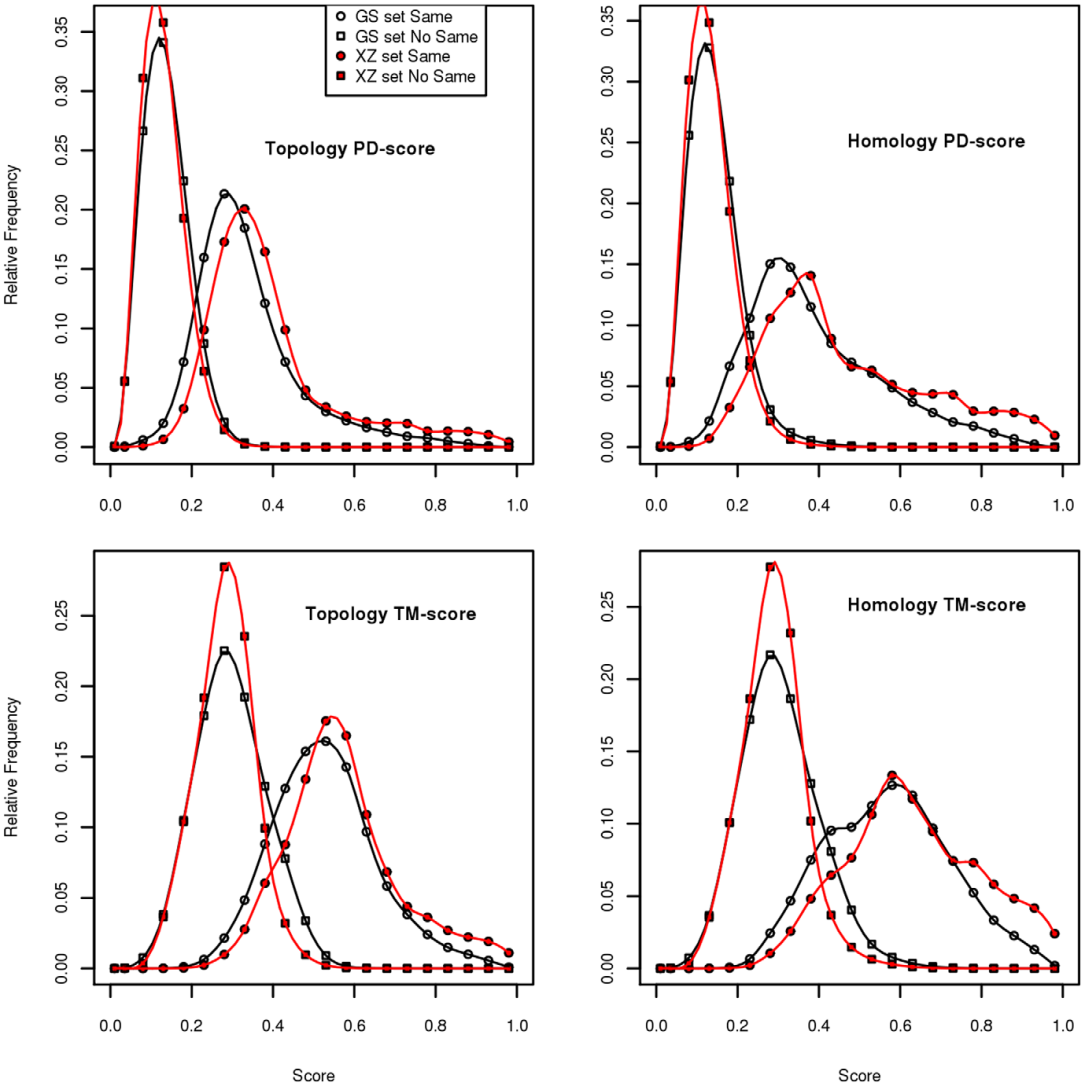
The frequencies of the scores for proteins in the same fold/homology and in different fold/homology for both benchmarks. Top: PD-scores, bottom: TM-scores, left: fold, right: homology.

Figure 7 displays the distribution of both scores for the two different sets. We shall provide quantitative measures later for the amount of overlap between the scores for same and different folds (or homologies).

We represent with 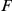 the event that both domains belong to the same SCOP fold and CATH topology. We use the values calculated by Xu and Zhang [14] using a large sample of SCOP and CATH databases: 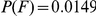 and 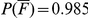.

Likewise, 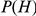 represents the probability that both domains are in the same CATH homology and SCOP superfamily. To estimate these values, we use the largest mapping (56.104 domains) between CATH and SCOP domains we know of (available at http://www.bio.ifi.lmu.de/webfm_send/1509) compiled by Csaba et al [18]. We calculate the number of domain pairs in the mapping where both domains are in the same CATH homology and SCOP superfamily, and the number of domain pairs where both domains are the different CATH homology and SCOP superfamily, relative to the sum of these two. We get 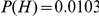 and 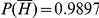.

**Figure 8:**
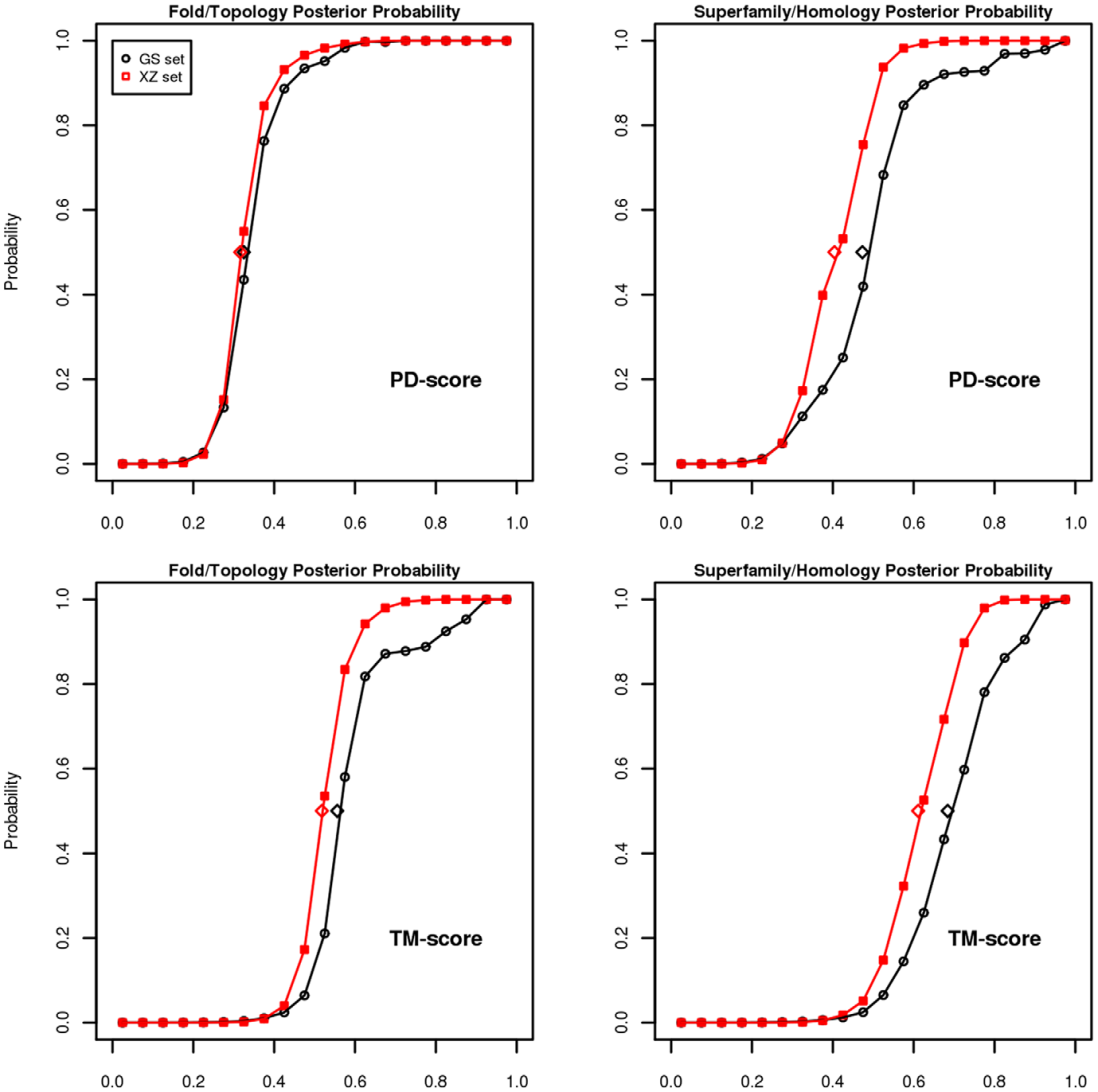
The posterior probability of proteins with a given score being in the same fold (or homology) for both sets XZ-sets and GS-sets. Top: for PD-scores. Bottom: for TM-scores. Left: fold level. Right, homology level. We can see that the phase transitions are clear, and apparently better for PDscores in homology discrimination, as confirmed in the figures below. The diamond marks indicate where the phase transitions occur.

In order to calculate the probability of being in the same fold given a PD-score, 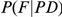, and given a TM-score, 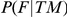, the Bayesian rule is applied over the probabilities 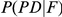 and 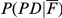, and similarly for the TM-scores, obtained from the score over the same and different fold sets. The same process is followed for the homology case. In Figure 8 we can see the posterior probabilities for a pair with a given score of being in the same fold (or homology), for the XZ-sets and the GS-sets. The GS-sets are difficult in the sense that there are several pairs with a different homology that receive high scores.

We define the score 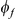 where the phase transition is produced as the value where 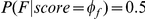, and the score 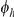, where 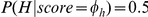. Above these thresholds the probabilities of being in the same fold (or homology) are above 0.5, and below the thresholds, the probabilities are below 0.5. The thresholds calculated are in Table 3. This includes the a posteriori error probabilities 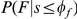 and 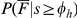, and the a priori error probabilities 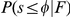 (probability of type II errors) and 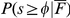 (probability of type I errors, i.e. P-values). All errors are calculated not only for both benchmark sets and but also for the homology discrimination case.

**Table 3:** A priori and a posteriori error probabilities for TM-align and ProtDeform, considering two benchmarks and both fold and homology levels.

	threshold			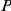 (type II errors)	P-value
	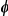	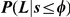	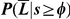	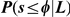	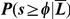
GS-PD-Topol.	**0.325**	**0.0086**	**0.2148**	**0.573836**	**0.001791**
GS-TM-Topol.	**0.556**	0.0094	0.2388	0.627692	0.001799
XZ-PD-Topol.	0.316	0.0057	0.1574	0.376840	0.001780
XZ-TM-Topol.	0.518	0.0058	0.1501	0.382491	0.001667
GS-PD-Homol.	**0.473**	**0.0075**	**0.2414**	**0.729508**	**0.000896**
GS-TM-Homol.	**0.684**	0.0080	0.3072	0.774336	0.001041
XZ-PD-Homol.	0.404	0.0051	0.1581	0.489770	0.000997
XZ-TM-Homol	0.611	0.0052	0.1945	0.498525	0.001260

The letter 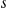 represents the score, 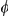, the score where the phase transition is produced and 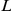, the fold or homology levels.

The table confirms the threshold 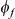 of around 0.5 suggested by Xu and Zhang [14] for TM-align at the topology level; it is 0.518 for the XZ-sets and 0.556 for the GS-sets. We would suggest the latter one because this set is more reliable. With respect to ProtDeform, we suggest 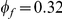 for fold/topology discrimination. In the superfamily/homology case, we found that 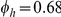 is a safe threshold when using TM-align, and 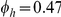 when using ProtDeform. We give more relevance to the Gold Standard set.

Let us finish this section by putting the a posteriori probabilities to use. One study that depends on the probabilities of the TM-align score is *On the origin and highly likely completeness of single-domain protein structures* by Zhang et al [19]. In this research, a set of virtual domains is randomly computer generated and compared to a set of compact PDB native structures. For a given domain in one set, the closest domain in the other set is found by TM-align and then a refined model is predicted by TASSER. In one test, it is said that the average TM-score for the templates found by TM-align is 0.47 and that the average TM-score of the predicted models for only the ten worst is 0.62. Unfortunately, the refined models are not calculated for all templates, nor are the standard deviations given. If we look at Figure 6 in [14] or our Figure 8, the probability of proteins with a TM-score of 0.47 being in the same fold is lower than 0.2. Nevertheless, for the known value of 0.62 for refined models, this probability is around 0.8. It is concluded that the virtual set is highly likely complete with respect to the protein universe.

Although their conclusion may be true, one problem that makes the completeness weak is that the average TM-scores of 0.47 and 0.62 are in the region around a TM-score of 0.5 where the phase transition in the a posteriori probability occurs. Even without knowing the standard deviations we can expect that part of the structures of one set have templates or models in the other set with a low probability of being in the same fold. Another problem is that in the context in which the best template is searched for in a domain library, we normally find higher quality alignments. For instance, Zhang et al. [10] (pag. 2306) reported that misfolded proteins from prediction tests are found to have close structure analogs in the PDB with an average RMSD = 3 Å and 87% of the residues aligned (the average TM-score seems to be above 0.6 in their Figure 4B), while the completeness study reports that single-domain proteins in the current PDB structural repertoire can be matched to the virtual structure library with an average RMSD of only 4 Å, 75% of the residues aligned and TM-score of 0.47. The last problem we see is that refined models were not calculated for all the templates, so we ignore the real TM-score improvement for the potential models that can be generated. In short, their assertion may be true but we think that it has yet to be shown conclusively that for each structure of one set in a given fold, a model that can be considered in the same fold with a high probability can be built from a structure taken from the other set.

### Classification performance

**Figure 9:**
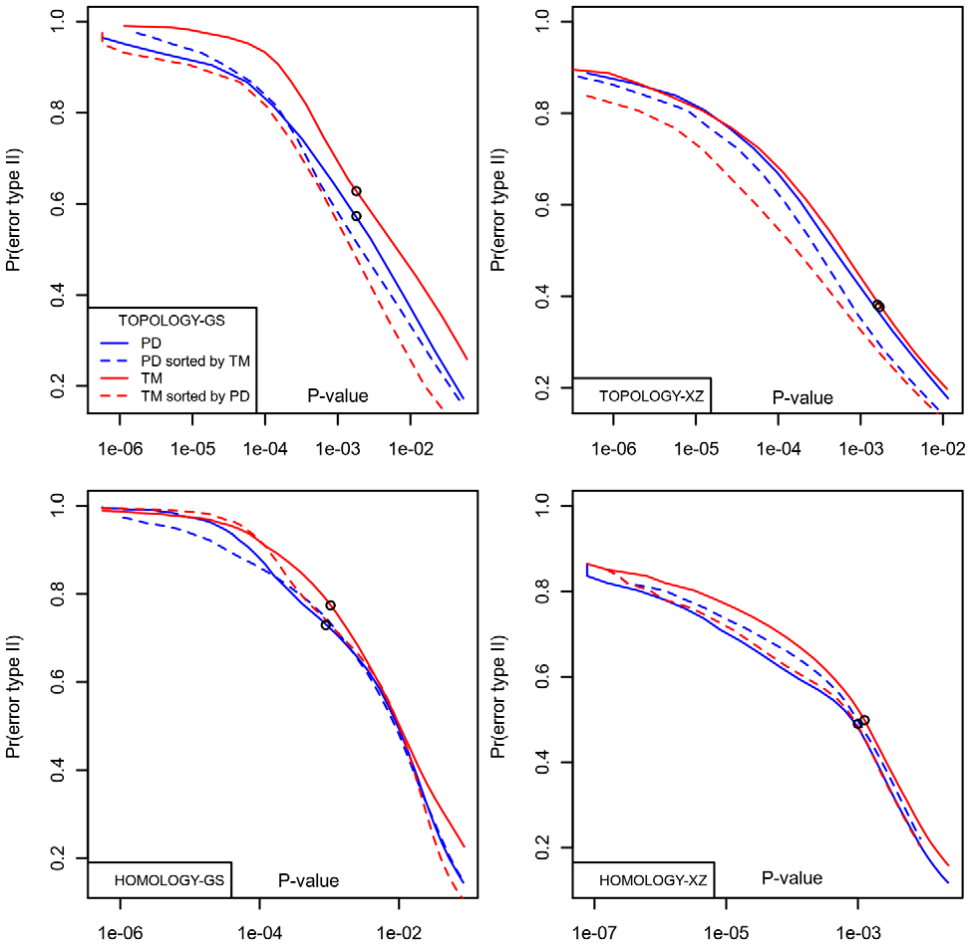
Prob(error type I) vs Prob(error type II) for TM-align and ProtDeform for both benchmark sets. Top panel: for the fold/topology level. Bottom panel: for the superfamily/homology level. The points mark the places where the a posterior probabilities are 0.5. From these points to the left, these probabilities are above 0.5. For the GS-sets, and also for the homology level, ProtDeform has lower probability of error type II at all probabilities of error type I (P-values); for the XZ-fold benchmark, the probabilities are similar. We can see a higher decision performance of ProtDeform over TM-align on three of the four tests with the fourth being similar. The dashed lines are re-scored tests. PD-scores always improve the TM-align results while TM-scores slightly improve the ProtDeform alignments for the Topology level.

The probability of the different type of errors is lower for ProtDeform than for TM-align for the GS-set, and at both topology and homology levels. For the XZ-test, TM-align is slightly better at the topology level, but at homology level, ProtDeform has fewer errors. The continuous lines in Figure 9 reveal the full relationship of the two types of errors and a higher decision performance of ProtDeform over TM-align on three of the four tests and a similar performance on the other one. This higher performance is confirmed by other types of quality measures. The probability, for example, that a query domain has its maximum score with a domain of the same fold (or homology) is calculated in Table 4 for all the domains in the XZ-sets, and GS-sets.

**Table 4:** Probabilities that a query domain has its maximum score with a domain in the same fold (or homology).

	speed	XZ-fold	GS-fold	XZ-hom.	GS-hom.
TM	**3.03**	0.982	0.8902	0.9424	0.8191
PD	2.84	**0.984**	**0.8978**	**0.9499**	**0.8373**

The first column indicates average program speed in pairs/s.

In order to try to separate the performance of the two aligners from the quality of the two scores, we carried out the following re-scoring test: the output alignment found by one of the aligners was evaluated by the other score. By doing so, we repeated the decision performance analysis for TM-align re-scored by the PD-score and ProtDeform re-scored by the TM-score. The curves with broken lines in Figure 9 show that at the topology level and on both benchmark sets the probabilities of errors are lower for the re-scored tests. On the other hand, at the homology level, on both benchmarks, TM-align re-scored with the PD-score outperforms native TM-align, while ProtDeform performance re-scored with the TM-score declines at least near where the a posterior probabilities are 0.5. However, the differences at this level do not seem important. We can see that the use of PD-scores always improves the classification performance while TM-scores improve only the topology classification. PD-scores greatly enhance the topology classification while TM-scores improve it to a lesser extent. Nevertheless it is remarkable that TM-scores improve ProtDeform performance at the Topology level, perhaps suggesting that ProtDeform or its score is more suited for Homology discrimination. Also, the TM-score maybe has a good potential for Topology classification and after a more effective alignment search, it would perform even better.

**Figure 10:**
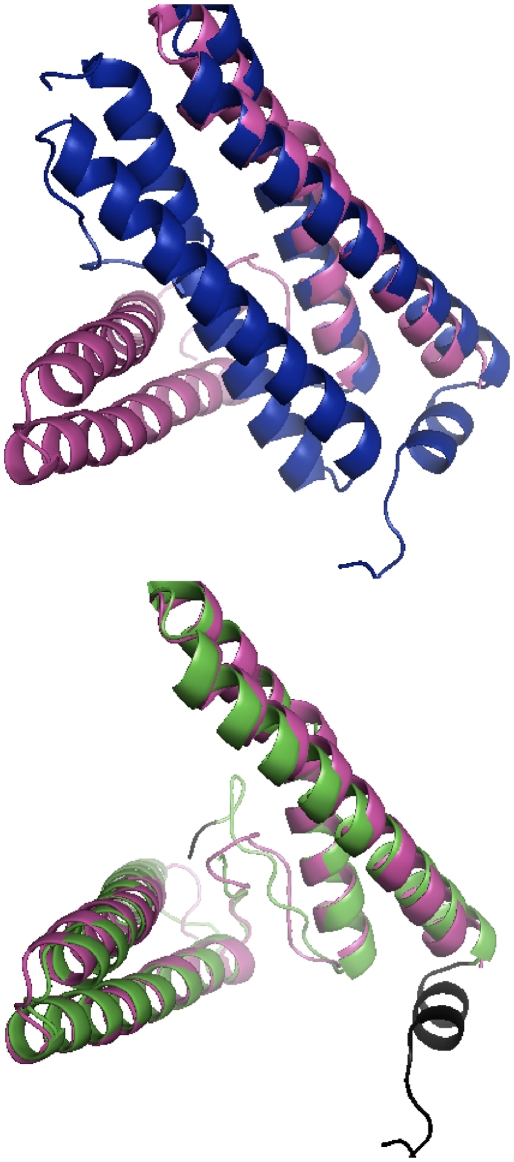
Two domains made of four long alpha helix structures, but in one of them, two of the helices are rotated. ProtDeform finds the way to align all four structures while TMalign can only align two of them. Top: Best superposition for the TMalign match. Bottom: Best superposition for the ProtDeform match. The pair is the ferritin-like protein domains d1j30a_ (a domain of surelythrin) and d1jgca_ (a domain of bacterioferritin).

One alignment example is the pair of ferritin-like protein domains d1j30a_ (a domain of surelythrin) and d1jgca_ (a domain of bacterioferritin) (both in SCOP family a.25.1.1 and CATH homology 1.20.1260.10). As shown in Figure 10, both contain 4 long 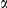-helices, but in d1j30a_ two of them are rotated. ProtDeform finds an alignment of 140 out 141 amino acids in the first domain, while TMalign only finds an alignment for two helices. ProtDeform is able to suggest different transformation for different parts of the protein while TMalign must reduce the alignment to find an accurate rigid transformation. Therefore, ProtDeform calculates a 0.93 probability of being in the same family while TMalign estimates a probability of only 0.05.

The first column of Table 4 also includes the average number of pairs per second per processor in a Dell computer with 8 processors. One can note that the speed of the programs is very similar.

## Discussion

We have carried out a detailed analysis of the statistical significance of PD-scores and TM-scores on several large benchmarks. We have confirmed some of the findings of Xu and Zhang [14], now using the Gold Standard benchmark for protein fold classification. We have broadened the analysis to the homology/superfamily level and shown that PD-scores have similar properties to TM-scores but are more reliable for classification. We have also proposed and calculated specific P-values for three different contexts.

For gapless alignment scores, we have shown that PD-scores also follow approximately an EVD, so that the P-values for PD-scores above 0.20 and the TM-scores above 0.27 are of the order of 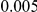, a relevant result, for instance, for alignments between a CASP folding model and a native structure. In fact, we have shown that around 20% of the CASP8 predictions have scores that do not reach this P-value. However, the fact that the distributions are very close to an EVD has no practical importance. With respect to length independence, both scores satisfy it but the TM-score is limited to the important range of medium-to-high scores.

For alignments calculated by TM-align, we confirmed that TM-scores above 0.56 correspond to a higher probability that the two domains are in the same fold, with a P-value of around 0.0018. In the ProtDeform case, the PD-scores above 0.33 have more chances of being from domains in the same fold, with a P-value of 0.0018, also. At the homology level, the thresholds are 0.68 and 0.47, with P-values of 0.001 and 0.0009, for TM-scores and PD-scores, respectively.

The relationship between probabilities for type I and type II errors, and another type of performance measure show that ProtDeform is a better discriminator of fold and homology than TM-align, with both programs running relatively fast (20 times faster than Dali [3]).

We estimated three different P-values (Figure 3) for the three different discrimination problems we are faced with. They are as follows: one is for the scores obtained for gapless alignments between domains of the same length; a second one is for the scores produced by optimisation programs on domains of different topology, and the third for the scores produced by the same programs on domains of different homology. Mixing these hypotheses can under-or over-estimate the significance of the scores seen, as was first calculated by Sierk and Pearson [20]. For example, Xu and Zhang [14] report a P-value(TM-score = 0.5) = 5.5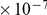, a far lower value than the correct probability, which is around 0.0017 (the probability of type I error, as seen in Table 3) for the context of fold discrimination by TM-align. In contrast, the former P-value is a good significance estimate with respect to gapless alignments produced under the context of folding.

In conclusion, we have found that PD-scores are length independent, discriminant and with a known significance. We wish to improve the score formula and find convincing arguments that make it useful for CASP.

## Materials and Methods

In this study, we analyse the PD-score in a coarser version than the one described in Rocha et al [3]. After reviewing the definitions, which include a new definition for backbone fragments, we describe the three benchmarks used to test the system.

### Basic definitions

To fix the notation, we denote complete protein structures by upper-case letters 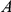, 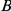. Each protein structure is described by its complete set of 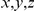 coordinates for all its atoms. We reduce this representation to the 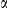-carbon backbone atoms 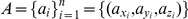, where 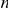 is the number of amino acids. They follow their own order in the protein chain, and each of them is called a protein site.

Let 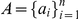 and 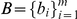 be two proteins. A *score matrix* for 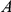 and 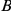 is a 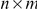-matrix 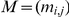, 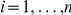, 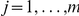. Intuitively, 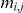 measures a likelihood of matching site 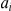 in 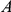 to site 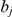 in 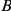. An *alignment* is a partial one-to-one order preserving function 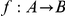. We denote by 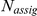 the number of pairs in 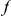.

### The fragments and their neighbourhoods

The new version of ProtDeform divides each protein backbone into fragments. If there are SSEs with fewer than 3 sites or backbone segments with no secondary structure with fewer than 3 sites then these shall appear as fragments. With the exception of this rare case, most fragments are of length 6 and they can go from 3 to 9 in such a way that in one fragment all amino acids are in the same secondary structure element (SSE) or in none, but there is no mixing of sites with different secondary structure. The method to calculate the fragments is as follows: assuming that the fragments are already defined for sites before a given one, the following 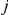 sites with a maximum of 9 that are in the same SSE or in none are considered. If 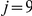 then the next fragment will be the first 6 sites starting with the given site and leaving 3 for the following fragment. If 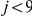 then the next fragment is made of the 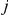 sites. The reason why there are at least 3 sites or more in a fragment is that when the previous fragment was calculated, 3 sites, if any, were left.

For each fragment 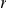, a neighbourhood 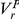 for protein 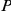 of 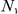 amino acids nearest the amino acids in the fragment is calculated (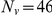, as it works best on our training set); the distance between an amino acid 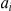 in a domain and a fragment is the shortest 3D distance between 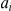 and the amino acids in the fragment. In the previous version, neighbours were calculated for sites instead of fragments. Then, given an alignment 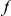 and the segments 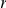 and 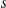, we define the local alignment 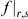 as the reduction of 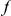 to the segments, formally,
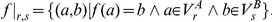



### The local transformations

We calculate the local transformation 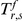 as the best rotation and translation from the 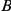 coordinate system to the 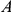 coordinate system, minimising the expression
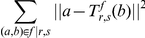
(when 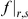 has fewer than three elements, we say that the transformation is not defined). In words, the transformation 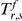 is the best one for the alignment 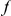 restricted to neighbours of the fragments 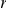 and 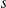. As stated above, this corresponds to a set of transformations coarser than the site transformations of the previous version.

Given a matching 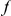, we calculate
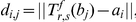
where 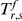 is the local transformation at the segments 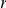 and 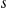, to which the sites 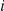 and 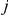 belong, respectively. We use the term
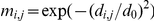
to calculate the score matrix as in the previous version. The value of 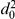 is still 11.5 Å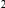.

### The algorithm

The algorithm consists of three main steps as in the previous version. First, we initialise a score matrix based on the first classifier in Matras [5]. Second, this score matrix is used to determine a match between sites by using a dynamic programming approach. Finally, this match is used to compute the set of local transformations, this time for each fragment pair rather than for each site pair. Each transformation maps a piece of the local structure of one protein onto the other. Armed with these transformations, we then compute a new score matrix based on the distances between the transformed sites. These last two steps are iterated until convergence or a maximum number of steps is reached. Convergence is reached when the matching does not change between successive steps.

The dynamic programming routine finds the matching 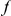 that maximises the value
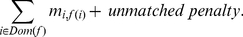
(1)The unmatched penalty is equal to the number of unmatched sites times a penalty value. We use the penalty value of 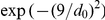, which corresponds, intuitively, to saying that an alignment of two sites with a distance between them greater than 9 Å is a poor alignment pair. We also avoid the alignments of site segments of a length below 6, i.e. any matched site will be with at least 5 other consecutive sites also matched.

### The PD-score

The final score for 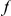 is computed as described in the original paper:
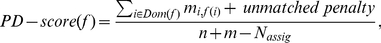
(2)where 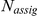 is the number of matched sites, and 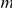 and 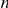 are the protein lengths.

### The Gold Standard benchmark

We use two benchmarks for protein classification and two for prediction score analysis. Our first benchmark for protein classification is the *Gold Standard* benchmark, a name given by its creators [18] because they want to set the standard for domain classification drawing on a precise consistent mapping between CATH and SCOP. They created two sets, one with domains in the same SCOP fold and CATH topology (GS-same-fold, 129,436 domain pairs) and one with domains in different SCOP fold and different CATH topology (GS-diff-fold, 1,740,476 domain pairs). In this way, we are more certain as to whether the domains in a given pair are really in the same structural class or not. Domains with 50% or more of sequence identity have been excluded to eliminate pairs easily classifiable. For the reasons just stated, this benchmark shall be taken as the most reliable among the ones considered in this study.

We then generate the set of pairs that are both in the same SCOP superfamily and CATH homology, the GS-same-homology (55,791 pairs), and the corresponding set of pairs in different SCOP superfamily and different CATH homology, the GS-diff-homology (1,814,121 pairs).

### The Xu-Zhang benchmark

The other benchmark for protein classification is the same consensus CATH-SCOP sets created for the TM-score analysis by Xu and Zhang [14]. Due to the protein identification disagreement between CATH and SCOP, a set has been created of 5,105 domain structures that do correspond to the same ID and identical protein regions in both CATH and SCOP (up to 90%). They do not exclude domains with a high sequence identity. Therefore, these sets, although larger, are easier than the ones in the previous benchmark. An all-to-all pairing is carried out, and we generate the set of domain pairs where both domains are in the same SCOP fold and CATH topology (named XZ-same-fold with 201,571 pairs), and also the set of pairs which are categorised into different SCOP fold and different CATH topology, (XZ-diff-fold, 12,497,203 pairs). Analogous to the previous benchmark, we also generate a consensus set for superfamily/homology analysis, the XY-same-homology set (38,778 pairs), and XY-diff-homology (12,921,651 pairs).

### The CASP8 predictions

We downloaded the 70,964 predictions made by the competitors in the CASP8 edition (http://predictioncenter.org/casp8). PD-scores and TM-scores were calculated for the trimmed domain predictions and the native structures under the identity alignment.

### The benchmark for gapless alignments

The set XZ-diff-fold is also used to create another benchmark for the significance analysis of alignments of domains of the same length and not in the same fold: for each domain pair, the shorter domain is superposed gaplessly to the longer domain, starting with the N-terminal, and sliding with leaps of 20 residues. An extra alignment, if needed, is generated aligning the C-terminals of the domains. This process creates six alignments on average for each domain pair for a total of 75,252,164 gapless alignments. Then, the PD-score and the TM-score are calculated for these simple alignments.
